# On the Use of Iron in Organic Chemistry

**DOI:** 10.3390/molecules25061349

**Published:** 2020-03-16

**Authors:** Arnar Guðmundsson, Jan-E. Bäckvall

**Affiliations:** 1 Department of Organic Chemistry, Arrhenius Laboratory, Stockholm University, SE-10691 Stockholm, Sweden; arnar.gudmundsson@su.se; 2 Department of Natural Sciences, Mid Sweden University, Holmgatan 10, 85179 Sundsvall, Sweden

**Keywords:** iron, organic synthesis, C-H activation, C-C coupling

## Abstract

Transition metal catalysis in modern organic synthesis has largely focused on noble transition metals like palladium, platinum and ruthenium. The toxicity and low abundance of these metals, however, has led to a rising focus on the development of the more sustainable base metals like iron, copper and nickel for use in catalysis. Iron is a particularly good candidate for this purpose due to its abundance, wide redox potential range, and the ease with which its properties can be tuned through the exploitation of its multiple oxidation states, electron spin states and redox potential. This is a fact made clear by all life on Earth, where iron is used as a cornerstone in the chemistry of living processes. In this mini review, we report on the general advancements in the field of iron catalysis in organic chemistry covering addition reactions, C-H activation, cross-coupling reactions, cycloadditions, isomerization and redox reactions.

## 1. Introduction

Iron is the most abundant element on Earth by mass and is used ubiquitously by living organisms [1]. The ability of iron to assume many oxidation states (from −2 up to +6) coupled with its low toxicity makes it an attractive, versatile and useful catalyst in organic synthesis. The wide range of oxidation states available for iron and its ability to promote single electron transfer (SET) allows it to cover a wide range of transformations. In low oxidation states, iron becomes nucleophilic in character and takes part in reactions such as nucleophilic substitutions, reductions and cycloisomerizations. At higher oxidation states, iron behaves as a Lewis acid, activating unsaturated bonds and at very high oxidation states (+3 to +5) iron complexes can take part in C-H activation. Due to iron’s central position in the periodic table, it can have the property of both an “early” and “late” transition metal and with the many oxidation states available, any type of reaction is, in principle, within reach. Iron cations also bind strongly to many N- and O-based ligands, and these ligands can replace phosphine ligands in iron chemistry.

As the atmosphere on Earth changed following the Great Oxidation Event about 2.4 billion years ago, ferrous (+2) iron complexes became less stable and ferric (+3) complexes became predominant [2]. Iron in its ferric oxidation state typically forms complexes that are water-insoluble like hematite (Fe_2_O_3_) or magnetite (Fe_3_O_4_), especially under basic conditions when exposed to air. This propensity of iron complexes to precipitate can be a hindrance for catalysis, although, despite the fact that ferric complexes are mostly water-insoluble at biological pH, iron is still the most common transition metal in living organisms and is indispensable for the chemical processes of life—oxygen binding, electron transport, DNA synthesis, and cell proliferation, to name only a few.

Modern catalysis has been dominated by noble transition metals, such as palladium, platinum, ruthenium and iridium, and these metals have been used in a wide range of reactions. The main advantage that noble transition metals have over their base counterparts is their preference for undergoing two-electron processes. They do, however, have significant drawbacks: high cost, non-renewable supply and/or precarious toxicological and ecological properties. These factors may not pose too much of a problem for academic research, but have profound implications for industry and for the future of sustainable chemistry. It is for these reasons that attention has shifted towards base transition metals like iron, copper and nickel. Iron is particularly well suited as it is the second most abundant metal in the Earth’s crust after aluminum and is consequently attractive economically and ecologically. Despite these advantages of iron, organoiron catalysts tend to suffer from serious drawbacks such as difficult synthetic pathways, lack of robustness, poor atom economy and low activity or enantioselectivity. Although circumventing these limitations will be necessary for iron catalysis to reach its full potential, base metal catalysis will no doubt gain importance in the future and it is reasonable to think that base metals such as iron will eventually supplant the traditional dominance of noble transition metals as the field matures. In recent times, the area of iron catalysis has exploded and Beller in 2008 and Bolm in 2009 declared that the age of iron has begun [3,4]. An intriguing outlook on the future of homogeneous iron catalysis was published in 2016 by Fürstner [5].

This review focuses on the recent advancements in iron catalysis pertaining to organic chemistry from 2016 to February 2020. An excellent and comprehensive review from 2015 by Knölker on all the types of iron-catalyzed reactions discussed in this review in addition to others can be consulted for the interested reader [6]. Other more specialized reviews may be found in each respective subsection. It should be mentioned that metathesis reactions are omitted from this review.

## 2. Iron in Organic Synthesis

Organoiron chemistry began in 1891 with the discovery of iron pentacarbonyl by Mond and Berthelot [7,8]. It was used sixty years later industrially in the Reppe process of hydroformylation of ethylene to form propionaldehyde and 1-propanol in basic solutions [9]. An important event was the discovery of ferrocene in 1951 [10], the structure of which was determined in 1952 [11,12] and led to the Nobel prize being awarded to Wilkinson and Fischer in 1973. The discovery of the Haber–Bosch process was an additional milestone in iron chemistry. The latter process uses an inorganic iron catalyst for the production of ammonia and sparked an agricultural revolution [13,14]. In modern organoiron chemistry, iron is used in a great number of diverse reactions, as will be apparent from this review, though perhaps, just as in the chemistry of life, its most ubiquitous role is in redox chemistry.

### 2.1. Addition Reactions

**Scheme 1:**
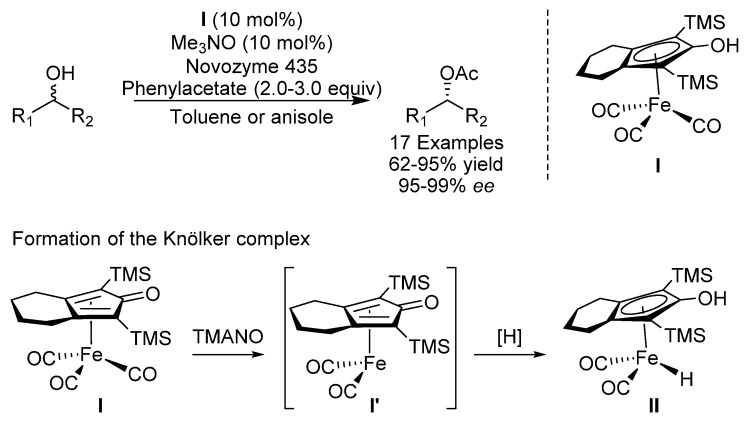
Bäckvall’s and Rueping’s DKR of *sec*-alcohols.

The first example of an iron-catalyzed racemization of alcohols was reported in 2016 with the use of an iron pincer catalyst [15]. Between 2016 and 2017, the groups of Bäckvall [16] and Rueping [17] independently reported the dynamic kinetic resolution (DKR) of sec-alcohols using a combination of iron catalysis for racemization and a lipase for resolution, which demonstrates a useful combination of enzyme and transition metal catalysis (Scheme 1). In one study, Knölkers complex (**II**) was used directly [17], and in the other study, a bench-stable precursor to Knölkers complex (**II**), iron tricarbonyl complex **I**, was used, which was activated through oxidative decarbonylation with TMANO to form coordinatively unsaturated iron complex **I’ [16]**. In the latter study, various benzylic and aliphatic esters could be produced in good to excellent yields with excellent *ee*. Two different enzymes, Candida antarctica lipase B (CalB/Novozyme 435) and Burkholderia cepasia (PS-C), could be used and the procedure could be reproduced on gram-scale. The group of Zhou also reported a related work using hexanoate as the acyl donor [18]. Rodriguez published a review on the synthesis, properties and reactivity of this interesting class of iron catalysts in 2015 [19].

**Scheme 2:**
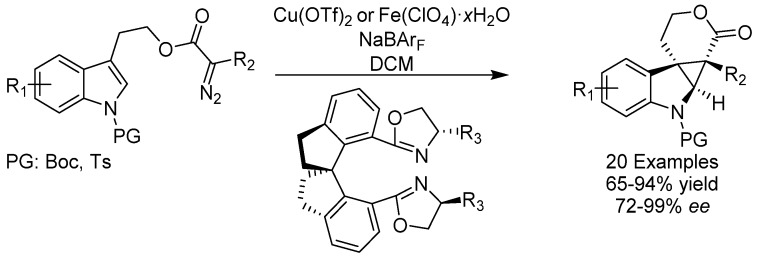
Zhou’s enantioselective cyclopropanation.

The indole ring is a ubiquitous heterocyclic motif in natural products and methods for constructing chiral polycyclic systems with indole skeletons has attracted considerable attention. In 2017, the group of Zhou reported the first intramolecular enantioselective cyclopropanation of indoles that was catalyzed either by iron or copper in the presence of a chiral ligand (Scheme 2) [20]. Many functional groups were tolerated and various cyclopropanated indoles were prepared in high to excellent yields and in almost all cases with excellent ee. The mechanism of the enhancement of the enantioselectivity is currently unknown, although the R_2_ group was found to be important and had to be different from hydrogen for the reaction to proceed.

**Scheme 3:**
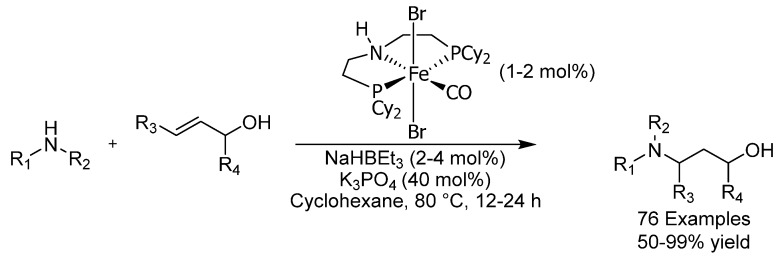
Wang’s iron-catalyzed anti-Markovnikov hydroamination.

Hydroamination of alkenes is an atom-economic approach and the amines produced are some of the most common functionalities found in fine chemicals and pharmaceuticals. Hydroamination of terminal alkenes typically gives the Markovnikov product selectivity, but in 2019 the group of Wang reported the first iron-catalyzed anti-Markovnikov addition of allylic alcohols [21]. For this purpose, an iron-PNP pincer complex was used. The reaction proceeds through a hydrogen-borrowing strategy where the iron complex temporarily activates the alcohol by dehydrogenation to the α,β-unsaturated carbonyl compound. The latter compound reacts with an amine to form an iminium ion, which undergoes conjugate additon at the β-position with another amine followed by hydrolysis and reduction to give the product. Various amines were produced in good yields with this method. Interestingly, hydroamidation could also be performed (Scheme 3).

**Scheme 4:**
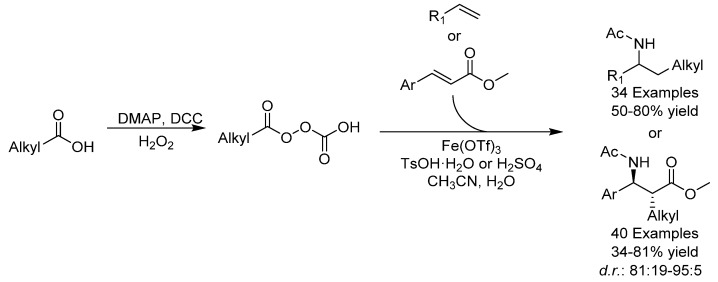
Bao’s carboamination.

C-C and C-N bonds are important bonds in organic chemistry and one of the most effective ways of creating these bonds simultaneously is through the carboamination of olefins. In 2017, the group of Bao reported the diastereoselective construction of amines and disubstituted β-amino acids through the carboamination of olefins (Scheme 4) [22]. Aliphatic acids were used as an alkyl source and nitriles as a nitrogen source. The protocol could be performed on a gram-scale and various carboamination products were obtained in good yields and excellent diastereoselectivity. The choice of acid was found to have a strong effect on the diastereoselectivity. TsOH was used for the carboamination of olefins, but for the carboamination of esters, binary and ternary acids had a more positive effect over monoacids, with H_2_SO_4_ giving the best result. The addition of the nitrile group was found, through Density Functional Theory (DFT) calculations, to be diastereoselectivity-determining, and hyperconjugation was proposed to account for the anti-selectivity.

**Scheme 5:**
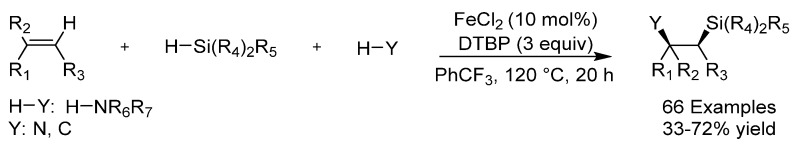
Luo’s silylation.

Organosilicon compounds have significant chemical, physical and bioactive properties and an example of these compounds is 1-amino-2-silylalkanes, which have, in recent times, emerged as candidates for pharmaceutical development. Silicon-containing compounds are generally made through hydrosylilation or dehydrogenative silylation, but in 2017 the group of Luo reported the first iron-catalyzed synthesis of 1-amino-2-silylalkanes through the 1,2-difunctionalization of styrenes and conjugated alkenes (Scheme 5) [23]. Di-tert-butyl-peroxide (DTBP) was used as an oxidant in the reaction. Amines, amides and carbon nucleophiles could be employed and delivered the corresponding products in mostly good yields. The reaction was proposed to proceed via a silicon-centered radical from oxidative cleavage of the Si-H bond followed by addition across the C=C bond and a N-H oxidative functionalization cascade.

### 2.2. C-H Bond Activation

**Scheme 6:**
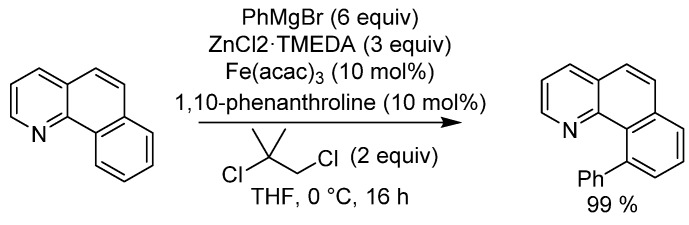
Nakamura’s C-H activation.

The first example of a C-H activation was a Friedel–Crafts reaction reported by Dimroth in 1902, where benzene reacted with mercury (II) acetate to give phenylmercury (II) acetate [24]. Later, in 1955, Murahashi reported the cobalt-catalyzed chelation-assisted C-H functionalization of (E)-N-1-diphenylmethaneimine to 2-phenylisoindolin-1-one [25]. A great advance in the field occurred in 1966 when Shilov reported that K_2_PtCl_4_ could induce isotope scrambling between methane and heavy water [26,27]. Shilov’s discovery led to the so called “Shilov system”, which remains to this day as one of the few catalytic systems that can accomplish selective alkene functionalizations under mild conditions. In 2008, the synthetic power of C-H activation was expanded to include organoiron catalysis by Nakamura in his arylation of benzoquinolines (Scheme 6) [28]. An excellent review on the subject of iron in C-H activation reactions by Nakamura was published in 2017 [29] and a review on oxidative C-H activation was published by Li in 2014 [30].

**Scheme 7:**
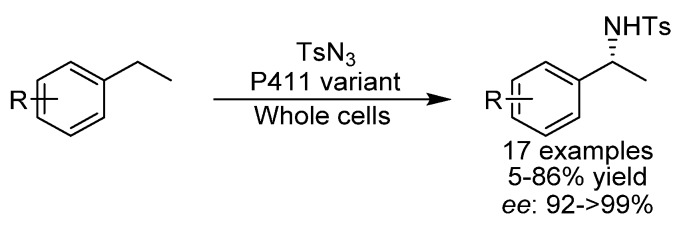
Arnold’s C-H amination.

The group of Arnold has in the past used engineered cytochrome P450, which is a type of enzyme that uses a heme cofactor, to enantioselectively α-hydroxylate arylacetic acid derivatives via C-H activation [31]. In 2017, they reported the directed evolution of cytochrome P450 monooxygenase, for enantioselective C-H activation to give C-N bonds (Scheme 7) [32]. It uses a variant of P411 based on the P450 monooxygenase which has an axial serine ligand on the haem iron instead of the natural cysteine. The method utilizes a tosyl azide as a nitrene source which generates an iron nitrenoid that subsequently reacts with an alkane to deliver the C-H amination product. The P411 variant has a turnover number (TON) of 1300, which is considerably higher than the best reported, to our knowledge, for traditional chiral transition metal complexes, which is a chiral manganese porphyrin with a turnover number of 85 [33]. A variety of benzylic tosylamines could be produced with excellent ees.

**Scheme 8:**
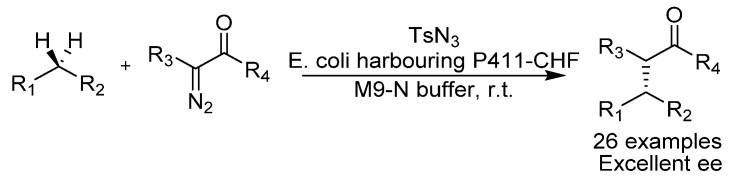
Arnold’s sp^3^ C-H activation.

In 2019, Arnold and coworkers extended their methodology using another variant of P411 in C-H alkylation using diazoesters (Scheme 8) [34]. The diazo substrate scope could be extended beyond ester-based reagents to Weinreb amides and diazoketones and gave the corresponding products with excellent *ee*s and with total turnover numbers (TTN) of up to 2330. These studies together show the potential for generating C-H alkylation enzymes that can emulate the scope and selectivity of Natures C-H oxygenation catalysts.

**Scheme 9:**
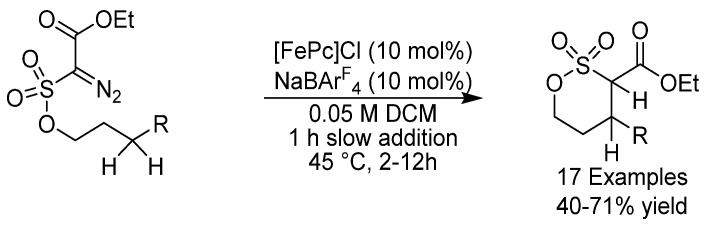
White’s isoelectronic carbene C(sp^3^)-H oxidation.

C(sp^3^)-H alkylation via an isoelectronic iron carbene intermediate was first reported in 2017 by the group of White using an iron phthalocyanine (Scheme 9) [35]. Iron carbenes generally prefer cyclopropanation over C-H oxidation, but, in this case, allylic and benzylic C(sp^3^)-H bonds could be alkylated with a broad scope. Mechanistic studies indicated that an electrophilic iron carbene was mediating homolytic C-H cleavage followed by recombination with the resulting alkyl radical to form the new C-C bond. The C-H cleavage was found to be partially rate determining.

**Scheme 10:**
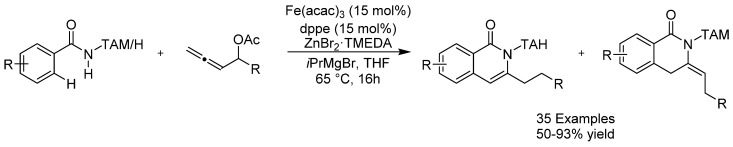
Ackermann’s allene annulation.

In 2018, the group of Ackermann reported on an allene annulation through an iron-catalyzed C-H/N-H/C-O/C-H functionalization sequence (Scheme 10) [36]. The mechanism was shown to involve an unprecedented 1,4-iron migration C-H activation manifold. Alkyl chlorides were tolerated under these reaction conditions, with no cross-coupling being observed. Various dihydroisoquinolones could be produced through the use of this method in excellent yields and the modular nature of the triazole group allowed for the synthesis of exo-methylene isoquinolones as well.

**Scheme 11:**
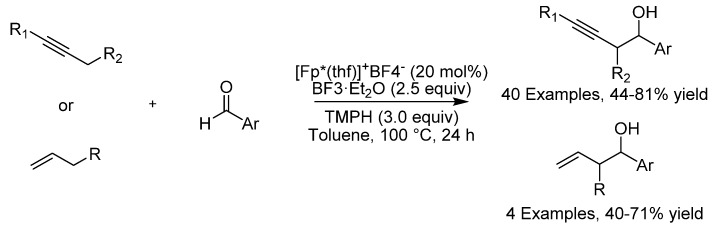
Wang’s α-C-H functionalization.

In late 2019, the group of Wang demonstrated an iron-catalyzed α-C-H functionalization of π-bonds in the hydroxyalkylation of alkynes and olefins (Scheme 11) [37]. Propargylic and allylic C-H bonds were functionalized with this method and a wide variety of homopropargylic and homoallylic alcohols could be produced in excellent yields, although with modest stereoselectivity. The key to the success of this approach is the fact that coordination of the iron catalyst to the unsaturated bond is known to lower the pKa of a propargylic or allylic proton from ≈38 and ≈43, respectively, to <10 [38]. An (α-allenyl)iron or (π-allyl)iron complex for propargylic or allylic complexes, respectively, is then formed in the presence of a base, which is utilized as the coupling partner.

**Scheme 12:**
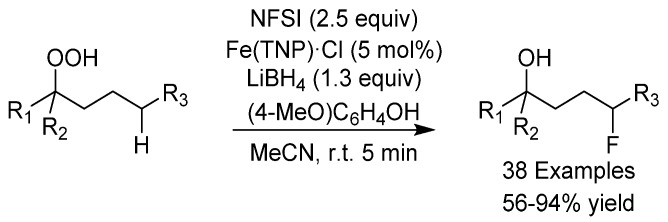
Liu’s C-H fluorination.

In 2018, the group of Liu reported the unprecedented iron(II)-catalyzed fluorination of C(sp^3^)-H bonds using alkoxyl radicals (Scheme 12) [39]. The procedure was applied to a wide range of substrates and it was found that a range of functional groups were tolerated, including halide and hydroxyl groups. N-fluorobenzenesulfonamide (NFSI) was used as the fluoride source and the substrate scope could be extended from fluorination to chlorination, amination and alkylation. The authors also demonstrated a one-pot application of their protocol starting from a simple alkane.

### 2.3. Cross-Coupling Reactions

**Scheme 13:**
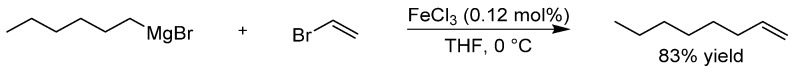
Kochi’s and Tamura’s original alkenylation.

Transition-metal-catalyzed cross coupling protocols have become an important tool in the organic chemist‘s arsenal. This area has been important in chemistry for about five decades, and in 2010 it received formal recognition when Richard Heck, Akira Suzuki and Ei-ichi Negishi received the Nobel prize for palladium-catalyzed cross-couplings in organic synthesis. Although a powerful technique, its applications have been dominated by the use of expensive palladium- and nickel-based catalysts, which are often toxic. The most common types of cross coupling reactions using iron are those involving Grignard reagents as the transmetalating nucleophile. The first example of an alkenylation of alkyl Grignard reagents with organic halides using iron(III) chloride was reported in 1971 by Kochi and Tamura (Scheme 13) [40]. A review on the subject of iron-catalyzed cross-coupling reactions with a focus on mechanistic studies was published in 2016 by Byers [41]. A more focused review on the use of iron-catalyzed cross-coupling for the synthesis of pharmaceuticals was released in 2018 by Szostak [42].

**Scheme 14:**
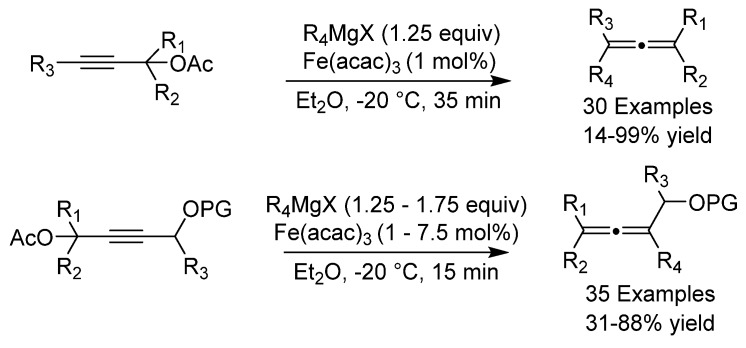
Bäckvall’s synthesis of substituted allenes and protected α-allenols from carboxylates.

In 2016, Bäckvall and coworkers reported the coupling of propargyl carboxylates and Grignard reagents using the environmentally benign Fe(acac)_3_ to synthesize substituted allenes and protected α-allenols (Scheme 14) [43,44]. The mild reaction conditions tolerate a broad range of functional groups (silyl ethers, carbamates and acetals) and could be applied to more complex molecules such as steroids. Tri and tetra substituted allenes were obtained in excellent yields, whereas the yield was found to drop for less substituted allenes. A variety of alkyl and aryl Grignard reagents could be applied and it was demonstrated that the protocol can be readily performed on a gram-scale.

**Scheme 15:**
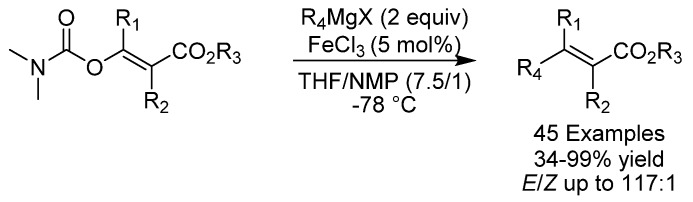
Frantz’ stereoselective synthesis of acrylates.

In 2016, the group of Frantz reported on a highly stereoselective iron-catalyzed cross coupling using FeCl_3_ to couple Grignard reagents and enol carbamates (Scheme 15) [45]. Many functional groups, such as ethers, silanes, primary bromides, alkynes and alkenes, were tolerated. In almost all cases, the yield and E/Z selectivity was excellent, with (E)-carbamates leading to (E)-acrylates and (Z)-carbamates leading to (Z)-acrylates. This study constitutes the only example so far of an iron-catalyzed cross-coupling, where an oxygen-based electrophile is favored over a vinylic halide (a Cl group at R_2_ in Scheme 15).

**Scheme 16:**
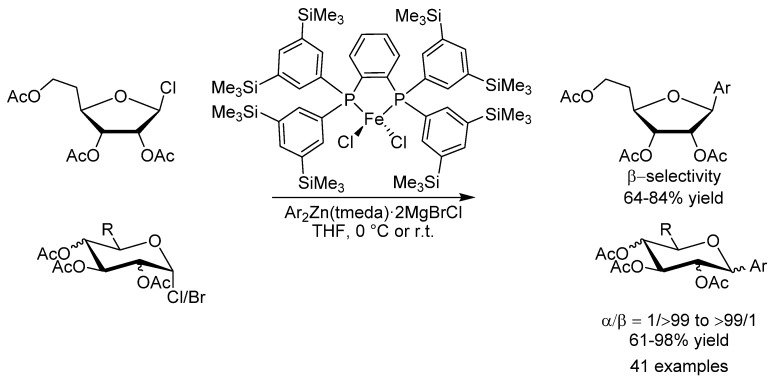
Nakamura’s diastereoselective synthesis of aryl C-glycosides using (Sciopp) FeCl_2._

Aryl C-glycosides are interesting pharmaceutical candidates because of their biological activities and resistance to metabolic degradation. In 2017, the group of Nakamura developed a highly diastereoselective iron-catalyzed cross-coupling of glycosyl halides and aryl metal reagents to form these compounds using FeCl_2_ in conjunction with a SciOPP ligand (Scheme 16) [46]. A variety of aryl, heteroaryl and vinyl metal reagents based on magnesium, zinc, boron and aluminium could be applied. The reaction was found to proceed through the generation and stereoselective trapping of glycosyl radical intermediates and represents a rare example of a highly stereoselective carbon-carbon bond formation based on iron catalysis.

**Scheme 17:**
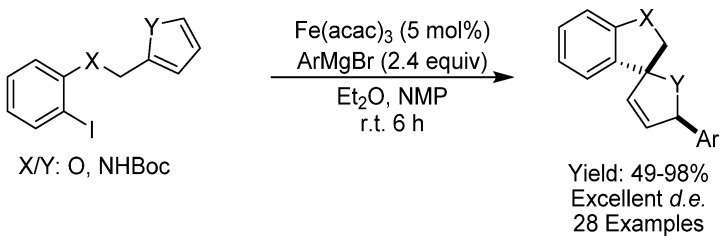
Sweeney’s cyclization.

Heterocyclic motifs are common in biologically active compounds and the presence of heteroatoms arranged around a quaternary carbon center often endows a certain spacial definition that can be useful, for example, in enhancing drug-binding. These types of spirocyclic motifs are most often generated through [2 + 3] cycloadditions, but, in 2017, the group of Sweeney developed an elegant cross-coupling cascade reaction to generate these motifs with inexpensive Fe(acac)_3_ as the catalyst directly from feedstocks chemicals directly available from plant sources (Scheme 17) [47]. The protocol delivered diastereomerically enriched nitrogen- and oxygen-containing cis-heterospirocycles and was applicable to substrates with typically sensitive functionalities like esters and aryl chlorides.

**Scheme 18:**
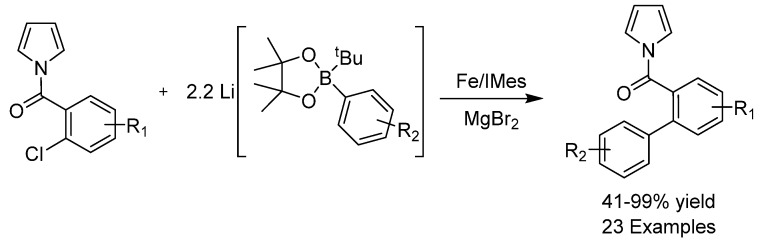
Bedford’s cross-coupling.

Palladium catalysts have traditionally been ubiquitous in cross-coupling reactions. Utilizing iron for the Suzuki coupling to provide simple biaryl compounds remained elusive until recently, when the group of Bedford showed that simple π-coordinating N-pyrrole amides on the aryl halide substrate could facilitate activation of the relatively unreactive C-X bond (Scheme 18) [48]. The use of an NHC ligand with the appropriate steric bulk proved crucial. A variety of biaryl products were obtained in excellent yields. This study demonstrates that iron-catalyzed Suzuki couplings to give biaryls are achievable, though to make the procedure general, efforts will have to be made to develop non-directed halide bond activation.

### 2.4. Cycloadditions

**Scheme 19:**
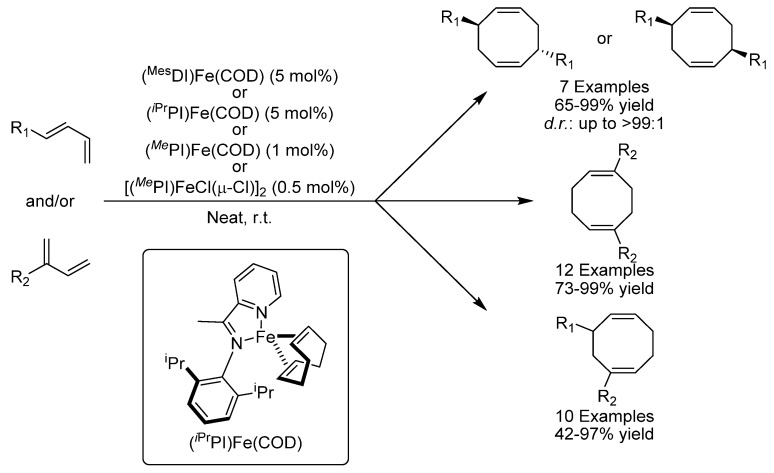
Chirik’s iron-catalyzed [4 + 4]-cycloaddition.

Medium-sized rings (7–11 membered) are important structural motifs with applications in the synthesis of polymers, fragrances and other specialty chemicals. Among these cyclic compounds, eight-membered rings are particularly useful for the synthesis of polyethylene derivatives. Metal-catalyzed [4 + 4]-cycloaddition of two butadienes to produce 1,5-cyclooctadiene is well established, although the use of 1,3-dienes is challenging owing to unwanted side-products including linear oligomers, [2 + 2]- and [4 + 2]-cycloadducts, as well as regio- and stereoisomeric [4 + 4] products. Catalyst design must be guided with these concerns in mind. Recently, the group of Chirik developed an iron-catalyzed [4 + 4]-cycloaddition of 1,3-dienes to access these compounds using (imino)pyridine iron bis-olefin and α-diimine iron complexes (Scheme 19) [49]. A wide variety of 1,5-cyclooctadienes could be produced in good to excellent yields and with controlled chemo- and regioselectivity. Kinetic analysis and Mößbauer spectroscopy provided evidence for a mechanism in which oxidative cyclization of the two dienes determines the regio- and diastereoselectivity.

**Scheme 20:**
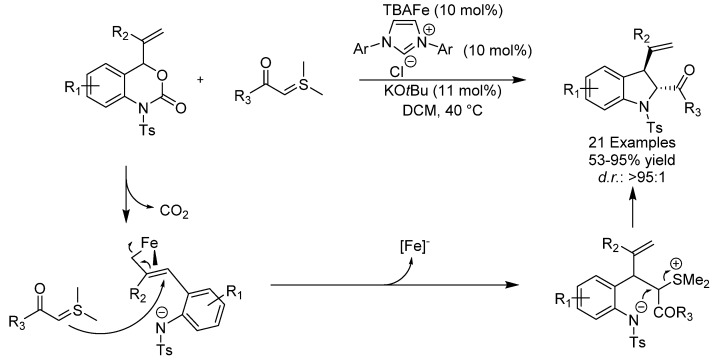
Xiaos’ [4 + 1]-cycloaddition.

Indolines are pharmaceutically significant aza-heterocycles and the first example of a synthesis of these compounds via an iron-catalyzed decarboxylative [4 + 1]-cycloaddition was described in 2016 by the group of Xiao. A wide range of functionalized indolines could be prepared from vinyl benzoxazinanones and sulfur ylides in good to excellent yields in high diastereoselectivity using the nucleophilic Bu_4_N[Fe(CO)_3_(NO)] (TBAFe) catalyst (Scheme 20) [50]. The authors postulated that the reaction proceeds via the formation of an electrophilic (π-allyl) iron complex, which would subsequently react with the sulfur ylide and undergo an intramolecular S_N_2 displacement of dimethyl sulfide by the tosyl amide anion. This study is noteworthy and constitutes the first example of the exploitation of an interesting reverse-electron-demand (π-allyl) iron species.

**Scheme 21:**
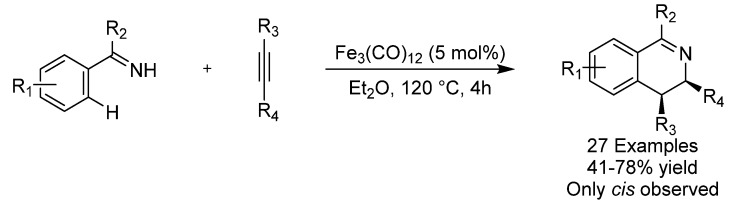
Wang’s [4 + 2] annulation/C-H activation.

Oxidative [4 + 2] annulations of imines and alkynes for the synthesis of isoquinolines are well established [51] but an interesting redox-neutral variant was reported in 2016 by the group of Wang involving an iron-carbonyl-catalyzed C-H transformation of an arene (Scheme 21) [52]. A wide variety of isoquinolines were isolated in good to excellent yield and only the cis-stereoisomer was observed. Mechanistic studies revealed that there was an essential synergy of dinuclear irons in the oxidative C-H addition and turnover-limiting H-transfer to the alkyne in the catalytic cycle.

**Scheme 22:**
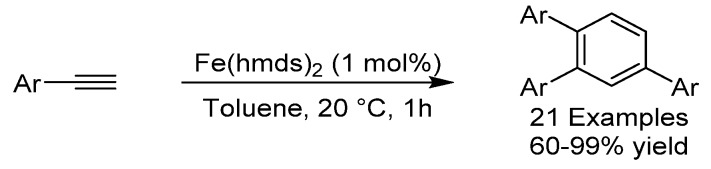
Jacobi van Wangelin’s cyclotrimerization.

Cyclotrimerization of alkynes mediated by transition metals is a key approach towards the synthesis of substituted aromatic compounds. In 2017, the group of Jacobi von Wangelin reported the iron-catalyzed trimerization of terminal alkynes in the absence of a reducing agent (Scheme 22) [53]. The approach was based on having a simple Fe (II) precatalyst and an internal bulky basic ligand which could mimic the effect of an external reductant. Terminal alkynes readily reacted, whereas internal ones did not, which is in accordance with the mechanistic postulate that the reaction is initiated through alkyne deprotonation. Good regioselectivity was observed with almost exclusive formation of the 1,2,4-substituted product over the 1,3,5-substituted and a wide variety of arenes were produced in mostly excellent yields.

### 2.5. Isomerizations

Iron-catalyzed isomerization of unsaturated alcohols to carbonyls was first reported by Emerson and Pettit in the 1960s [54]. In this study, butadiene-(tricarbonyl)iron complexes were exposed to strong acids to form their corresponding π-allyl iron complexes. When these were reacted with water, η^2^-(allyl alcohol)iron complexes formed, which isomerized to the enol-iron complex. Upon decomposition, butanone was obtained.

**Scheme 23:**
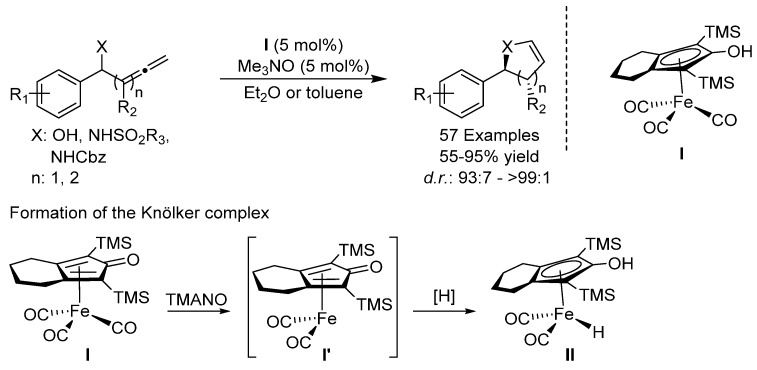
Bäckvall’s and Rueping’s cycloisomerizations.

Between 2017 and 2019, the groups of Bäckvall [55,56] and Rueping [57,58] independently reported the iron-catalyzed cycloisomerization of allenols and allenic sulfonamides using iron tricarbonyl complex **I** (Scheme 23). An analogous ruthenium-catalyzed cyclization of α-allenols had previously been reported by Bäckvall [59]. In these protocols, **I** is activated by TMANO to form the active catalyst **I’.** The protocols have a wide scope and deliver the corresponding heterocycles in excellent yields. Interestingly it was found that through the introduction of a substituent in the R_2_ position of the allene, the reaction was made diastereoselective, giving the trans-product predominantly in the case of dihydrofurans or exclusively in the case of dihydropyrroles [54,55]. The origin of the diastereoselectivity, revealed through DFT calculations, was the participation of the non-innocent cyclopentadienyl ligand of the catalyst lowering the transition state, leading to the trans product.

**Scheme 24:**
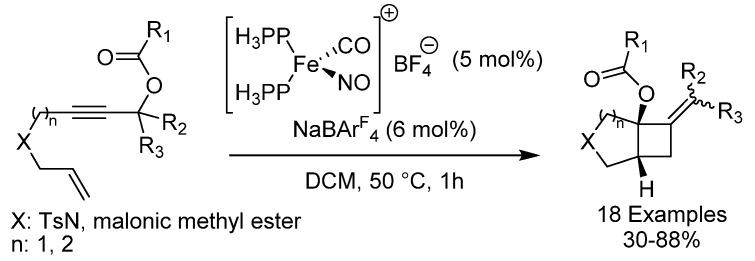
Plietker’s cycloisomerization of enynes.

According to the Woodward–Hofmann rules, [2 + 2] cycloadditions of two olefins are photochemically allowed, but thermally forbidden [60]. Due to this restriction, photochemical [2 + 2] cycloaddition between two olefins is the most common strategy to form cyclobutanes. In 2018, the group of Plietker reported an unusual cycloisomerization of enynes using a cationic iron nitrosyl catalyst (Scheme 24) [61]. Their 1,6- and 1,7-enyneacetates were expected to isomerize to allenes, but instead isomerized to their corresponding bicyclo [3.2.0] or [4.2.0] alkylamides in good to excellent yield under mild conditions. The reaction tolerates esters, amides and halides, and afforded the products generally in good to high yields.

**Scheme 25:**
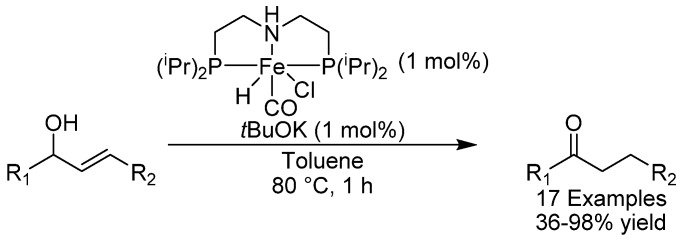
de Vries’ allylic alcohol isomerization.

Iron carbonyl complexes have been used in the isomerization of allylic alcohols, but a major drawback associated with the use of these complexes is the fact that they require UV light for activation and are thus not amenable to industrial synthesis [62]. In 2018, the group of de Vries reported the use of a pincer PNP-iron complex for the isomerization of allylic alcohols and found it to be an excellent racemization catalyst in conjunction with tBuOK (Scheme 25) [63]. Both benzylic and aliphatic allylic and homoallylic alcohols could be isomerized in mostly excellent yields. The sterically hindered trans-sobrerol could be reduced as well, although in a modest yield of 36%. A two-step hydrogen borrowing mechanism involving dehydrogenation-hydrogenation isomerization was proposed on the basis of DFT calculations.

**Scheme 26:**
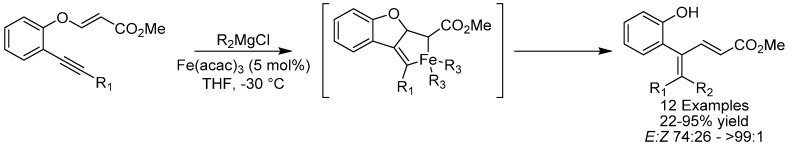
Fürstner’s cycloisomerization/cross-coupling cascade reaction.

An elegant cascade reaction involving cross-coupling and cycloisomerization forming two C-C bonds and highly functionalized 1,3-dienes with a stereodefined tetrasubstituted alkene unit, which is difficult to obtain by other means, was developed in 2016 by the group of Fürstner (Scheme 26) [64]. A series of X-ray structures showed that the substituent delivered by the Grignard reagent and the transferred alkenyl substituent were on the same side of the central tetrasubstituted alkene unit. Any loss of stereoselectivity is likely the result of a secondary isomerization process. Many variously substituted 1,3-dienes could be produced with this method in good to excellent yield and enantioselectivity and both primary and secondary Grignard reagents could be used.

### 2.6. Redox Reactions

Redox chemistry is what iron has traditionally been most known for, due to its multiple possible oxidation states. It is a very important field, not only for organic synthesis but also for biochemistry and industrial applications. A review on the use of iron (and other base metals) in borrowing hydrogen strategies was released by Morrill in 2019 [65]. A review on iron in reduction and hydrometalation was published in 2019 by Darcel [66].

One of the main bottlenecks in synthetic fuel production powered by sunlight or electricity is the oxidation of water. The use of abundant and environmentally benign metals for this purpose is desireable and, to this end, the group of Masaoka, in 2016, reported the use of a pentanuclear iron catalyst [Fe^II^_4_Fe^III^(μ_3_-O)(μ-L)_6_]^3+^ ((L = 3,5-bis(2-pyridyl)pyrazole) [67]. A multinuclear core typically gives rise to a redox flexibility that would be expected to be favourable in a reaction involving multi-electron transfer, such as water oxidation, as is known from enzyme examples in nature such as photosystem II in plants where Mn_4_Ca clusters are used. The turnover frequency was found to be 1900 per second, which is about three orders of magnitude greater than that of other iron-based systems, but despite the advantage of this system, it required a H_2_O/MeCN mixture and a large overpotential of 0.5 V, preventing it from practical use. These findings do, however, clearly indicate that efficient water oxidation protocols can be developed based on multinuclear iron catalysts.

**Scheme 27:**
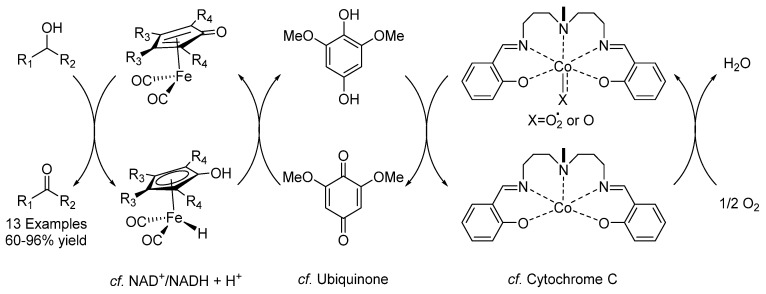
Bäckvall’s biomimetic oxidation of alcohols.

Oxidation reactions are of fundamental interest in organic chemistry, and an ideal oxidant to use in such processes is O_2_. To this end, the group of Bäckvall in 2020 reported the first example of a biomimetic oxidation of alcohols (Scheme 27) [68]. Various primary and secondary alcohols could be oxidized to their corresponding aldehydes and ketones with this method in good to excellent yields. The process was inspired by the electron transport chain used in living organisms and involves coupled redox processes that lead to a low-energy pathway through a stepwise oxidation using electron transfer mediators (ETMs) with O_2_ as the terminal oxidant. An iron(II)-catalyst was used for this purpose, which exhibited surprising stability in the presence of O_2_, in addition to a benzoquinone-derivative and a Co(salen)-type complex as ETMs.

**Scheme 28:**
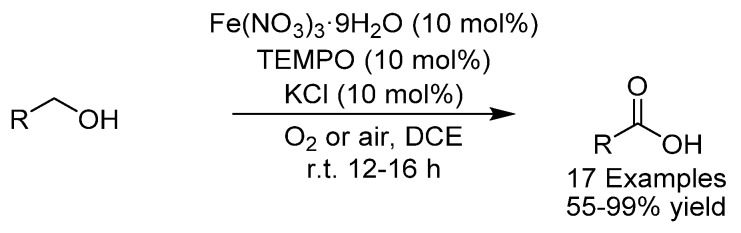
Ma’s iron-catalyzed aerobic oxidation of alcohols.

The oxidation of alcohols to carboxylic acids typically involves the use of stoichiometric amounts of toxic oxidants, such as KMnO_4_ or CrO_3_. In 2016, the group of Ma developed an iron-catalyzed aerobic oxidation of alcohols to carboxylic acids to circumvent this problem (Scheme 28) [69]. A wide variety of alcohols (and aldehydes) could be oxidized to their corresponding carboxylic acids and synthetically useful groups such as halides, alkynes, olefins, esters and ethers were tolerated. The synthetic application of this protocol was also demonstrated through the first total synthesis of the naturally occurring allene, phlomic acid, and in a 55 g synthesis of palmitic acid.

**Scheme 29:**
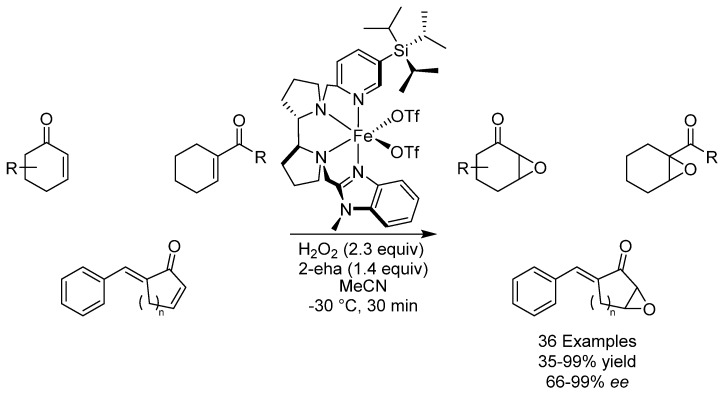
Costas’ enantioselective epoxidation.

Cyclic α-epoxide enones are structures found routinely in natural products and are also useful synthons. Producing these compounds through enantioselective epoxidation is notoriously difficult. In 2016, the group of Costas reported the first iron-catalyzed and highly enantioselective epoxidation of these compounds using a bulky tetradentate iron catalyst (Scheme 29) [70]. The reaction was found to proceed via the generation of an electrophilic oxidant, which is unusual for this class of reactions, where nucleophilic oxidants usually account for the reaction through a Weitz–Scheffer-type mechanism. 2-Ethylhexanoic acid (2-eha) was found to be necessary as an additive to help activate H_2_O_2_. Various epoxides could be produced in mostly excellent yields and excellent *ee*s.

**Scheme 30:**
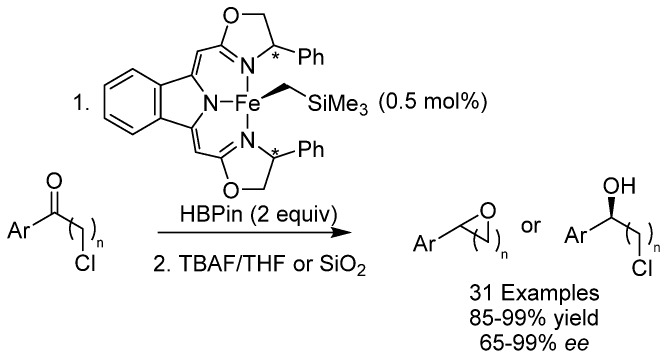
Gade’s enantioselective reduction of functionalized ketones.

A number of pharmaceutically active compounds for psychiatric therapy or cardic arrythmias have optically active alcohols or oxaheterocycle motifs. The standard methods of synthesizing these compounds rely on expensive and partly toxic noble metals and it would thus be advantageous to employ base metals for this purpose. In 2018, the group of Gade developed an enantioselective reduction of functionalized ketones to form these compounds (Scheme 30) [71]. A chiral pincer “boxmi”-type iron catalyst with a low catalyst loading of 0.5 mol% was used which provided access to a broad range of functionalized halohydrins, oxaheterocycles and amino alcohols in excellent yields and mostly excellent *ee*s. The protocol could also be performed on a gram scale to form a chiral halohydrin that forms the basis for the preparation of the antidepressant (R)-fluoxetine.

**Scheme 31:**
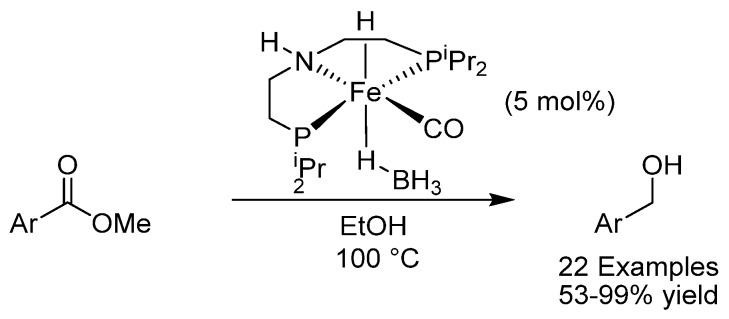
de Vries’ transfer hydrogenation of esters.

Transfer hydrogenation processes are attractive alternatives to classical catalytic hydrogenation since the use of high pressure and expensive reactors can be avoided. The group of de Vries in 2019 reported the base-free transfer hydrogenation of esters using EtOH as the hydrogen source (Scheme 31) [72]. It is interesting to note that EtOH can be used as the reductant, which is usually a problem, since the acetaldehyde formed can potentially deactivate the catalyst by decarbonylation. Various aliphatic and aromatic esters, including lactones, could be reduced to their corresponding alcohols in good to excellent yields. In addition the reaction could be scaled up tenfold. Similar iron-based protocols using a base are known to reduce C=C bonds [73], but in this base-free protocol, esters were selectively reduced. One important topic in green chemistry is the recycling of polymers by conversion to their monomers. This study shows the first transfer-hydrogenation-catalyzed depolymerization of a polyester (Dynacol 7360, made from adipic acid and 1,6-hexanediol), demonstrating the possibility of using iron-catalysis for the recycling of plastic waste.

**Scheme 32:**
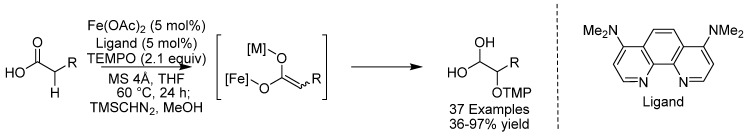
Ohshima’s α-oxidation of carboxylic acids.

Simple carboxylic acids are excellent carbonyl donors for functionalized carboxylic acid derivatives. Generating the enolate, enediolate, of a carboxylic acid is, however, quite challenging, due to the low acidity of the α-proton. The Brønsted acidity of the carboxylic acid is a further complication for the deprotonation of the α-proton, and therefore two equivalents of a strong base such as LDA are normally required for efficient enolization. In 2020, the group of Ohshima reported the α-functionalization of carboxylic acids through an iron-catalyzed 1e^-^ radical process [74]. The use of molecular sieves was found to be crucial in suppressing undesired decarboxylation and the alkali metal salt component of the sieves (sodium or potassium carboxylate) was found to play a crucial role in promoting the reaction. The reaction had wide scope and functional groups typically sensitive to oxidation conditions, such as primary benzylic alcohols and thioethers were tolerated. This study constitutes the first example of the generation of redox-active heterobimetallic enediolates from unprotected carboxylic acids under catalytic conditions (Scheme 32).

## 3. Conclusions

This review has highlighted recent advances in iron-catalysis with respect to organic synthesis. This field has expanded greatly in recent years and giving a detailed account of each reaction category is an impossible task for a short review of this type. Herein, only key representative examples are given, and interested readers are advised to look into the reviews referenced throughout the text.

As is clear from the examples given in this review, iron complexes are capable of catalyzing a wide range of reactions in organic synthesis. This potential of iron catalysis has not been fully realized, however, and noble transition metal protocols continue to define the state of the art when it comes to transition metal catalysis. The field is still in its infancy, but it is fair to say that iron (and other base metals) may very well challenge this dominance of noble transition metals in the foreseeable future.
